# Clinical correlates of movement disorders in adult Niemann-Pick type C patients measured via a Personal KinetiGraph

**DOI:** 10.1007/s10072-022-06308-0

**Published:** 2022-08-09

**Authors:** Shaddy El-Masri, Charles B. Malpas, Andrew Evans, Mark Walterfang

**Affiliations:** 1 Royal Melbourne Hospital, Parkville, VIC Australia; 2 Department of Neurology, Royal Melbourne Hospital, Parkville, Australia; 3 Department of Medicine, Royal Melbourne Hospital, The University of Melbourne, Melbourne, Australia; 4 Melbourne School of Psychological Sciences, The University of Melbourne, Melbourne, Australia; 5 Neuropsychiatry Unit, Royal Melbourne Hospital, Parkville, VIC 3050 Australia; 6 Melbourne Neuropsychiatry Centre, University of Melbourne and North Western Mental Health, Parkville, Australia; 7 Florey Institute of Neuroscience and Mental Health, Parkville, Australia

**Keywords:** Niemann-Pick type C, Personal KinetiGraph, Dyskinesia, Dystonia

## Abstract

**Background:**

Niemann-Pick type C (NPC) is an autosomal recessive progressive neurodegenerative disorder caused by mutations in the NPC1 or NPC2 genes. Patients with this disorder have variable phenotypic presentations that often include neuropsychiatric manifestations, cognitive decline, and movement disorders. There is considerable interpatient variation in movement disorders, with limited quantitative measurements describing the movements observed. Objective measurements using wearable sensors provide clinically applicable monitoring of patients with Parkinson’s disease, and hence may be utilized in patients with NPC.

**Objective:**

To explore the relationship between objective measurements of movement obtained via the use of the Personal KinetiGraph (PKG) with the clinical information obtained via questionnaires and clinical rating tools of patients with Niemann-Pick type C.

**Methods:**

Twelve patients with Niemann-Pick type C were recruited who wore the PKG for 6 days during regular activities. A 6-day output was provided by the manufacturer, which provided bradykinesia (BK) and dyskinesia (DK) scores. BK and DK scores were further divided into their interquartile ranges. A fluctuation score (FDS), percentage time immobile (PTI), and percent time with tremors (PTT) were also provided. Clinical assessments included Abnormal Involuntary Movement Scale (AIMS), Epworth Sleepiness Score (ESS), Falls, Neuropsychiatric Unit Assessment Tool (NUCOG), Parkinson’s disease questionnaire (PDQ), and modified Unified Parkinson’s Disease Rating Scale (UPDRS) which were performed over telehealth within 2 weeks of PKG use. Pearson’s correlation analyses were utilized to explore the relationship between DK and BK quartiles and clinical measures.

**Results:**

We found bradykinesia to be a feature among this cohort of patients, with a median BKS of 22.0 (7.4). Additionally, PTI scores were elevated at 4.9 (8.2) indicating elevated daytime sleepiness. Significant correlations were demonstrated between BK25 and Falls (*r* =  − 0.74, 95% CI = [− 0.95, − 0.08]), BK50 and Falls (*r* =  − 0.79, 95% CI = [− 0.96, − 0.19]), and BK75 and Falls (*r* =  − 0.76, 95% CI = [− 0.95, − 0.11]). FDS correlated with PDQ (*r* =  − 0.7, 95% CI = [− 0.92, − 0.18]), UPDRS IV (*r* =  − 0.65, 95% CI = [− 0.90, − 0.09]), UPDRS (*r* =  − 0.64, 95% CI = [− 0.9, − 0.06]), and AIMS (*r* =  − 0.96, 95% CI = [− 0.99, − 0.49]). DK25 in comparison with NUCOG-A (*r* = 0.72, 95% CI = [0.17, 0.93]) and DK75 in comparison with NUCOG (*r* = 0.64, 95% CI = [0.02, 0.91]) and NUCOG-A (*r* = 0.63, 95% CI = [0.01, 0.90]) demonstrated significant correlations. Additionally, duration of illness in comparison with PTI (*r* = 0.72, 95% CI = [0.22, 0.92]) demonstrated significance.

**Conclusions:**

Utilization of PKG measures demonstrated that bradykinesia is under recognized among NPC patients, and the bradykinetic patients were less likely to report concerns regarding falls. Additionally, the FDS rather than the DKS is sensitive to the abnormal involuntary movements of NPC—reflecting a differing neurobiology of this chorea compared to levodopa-induced dyskinesias. Furthermore, dyskinetic individuals performed better in cognitive assessments of attention which may indicate an earlier timepoint within disease progression.

## Introduction

Niemann-Pick type C (NPC) is a rare autosomal recessive progressive neurodegenerative disorder caused by mutations in the NPC1 or NPC2 genes, resulting in abnormal endosomal-lysosomal trafficking and subsequent accumulation of lipid-filled lysosomes within tissues [1, 2]. Phenotypic presentation varies between neonates and adults, but is characterized in the latter by psychiatric symptoms, cognitive decline, and ataxia [3–5], with earlier identification required to prevent deterioration with the emergence of demonstrated illness-modifying therapies [6, 7]. There is significant interpatient variation in the progression of movement disturbance—primarily ataxia—associated with NPC [8].

The Personal KinetiGraph (PKG) is a movement recording system validated in the routine care of patients with Parkinson’s disease [9]. The device was pre-empted by the ongoing pharmacologic therapeutic interventions aimed at motor symptom control that is dependent on clinical observation during frequent clinical assessment. The ability to provide continuous objective measurements of tremor, bradykinesia, and dyskinesia facilitates the monitoring of effects of the pharmacologic interventions in patients with Parkinson’s disease.

The PKG resembles a wristwatch preferably worn on the most affected arm. It contains a battery, accelerometer, memory, optional reminders for medication time administration, and a capacitive sensor that detects removal of the watch from the wrist. It is a wearable sensor system that provides objective ratings into the features of PD, including bradykinesia, dyskinesia, tremor, and fluctuations. In addition, it also measures sleep-related parameters [10], and provides medication dosing reminders for patients. The system has two algorithms that provide movement likelihood scores for dyskinesia or bradykinesia—the dyskinesia score (DKS) and the bradykinesia score (BKS) [9]. The fluctuating and dyskinesia score (FDS) was developed as a summary score of motor fluctuations and dyskinesias [11]. It represents the logarithm of the sum of the interquartile range of BKS and DKS during the recording period and has been shown to distinguish clinical fluctuators from non-fluctuators [12–14]. The PKG also measures immobility (percentage time immobile or PTI) as a surrogate marker of daytime sleep [15]. Finally, a percentage time tremor (PTT) score is provided, and is defined as the percentage of 2-min periods during 0900–1800 that contained tremors, which is likely to be present if the PTT score > 1% [16].

In Parkinson’s disease, output scores have been demonstrated to correlate with the Unified Parkinson’s Disease Rating Scale part III (UPDRS-III) and the Abnormal Involuntary Movement Score (AIMS) [9]. Furthermore, it provides the clinician with data captured during regular activities of daily living in their home environment and hence can provide objective information that can be interpreted in patients with Parkinson’s disease. In adult patients, NPC presents with a range of movement disturbance, including ataxia, dystonia, chorea, and myoclonus [3–5], as well as sleep disorders [17]. Thus, the measures captured by the PKG could thus provide useful objective measures on the movement disturbance in adults with NPC.

This study investigated the relationship between the various objective parameters obtained via the PKG and clinical assessments obtained from a cohort of adult patients with NPC to allow characterization of movement disorder in this patient group.

## Methods

A cohort of individuals with NPC and aged > 18 years consenting to protocols approved by the Melbourne Health Research and Ethics Committee (MH2012.066) were included in the study.

All subjects were de-identified prior to analyses.

### The PKG

The PKG system design, measurement, and output have previously been described in detail and have been extensively validated in patients with Parkinson’s disease. The device was worn on the most affected side for at least 6 days and, when completed, was returned to the manufacturer for data extraction and analysis. An output of the bradykinesia (BKS) and dyskinesia scores (DKS) was obtained every 2 min during use. Thus, between 0900 and 1800 each day, there are 270 DKS and BKS values, and 1620 over the course of 6 days. The median of these scores is referred to as the median BKS and DKS for the individual. Hence, the BKS value generated is based on an algorithm that recognizes bradykinesia as having fewer movements when compared with normal subjects that are made with lower acceleration and amplitude and with longer intervals between movements, whereas dyskinesia is recognized as having movements of normal amplitudes and acceleration, but with shorter periods without movement [9]. Furthermore, dyskinetic movements are characterized as having larger acceleration values in movements that are slower than the mean in comparison to normal subjects who performed these slow movements with a normal acceleration [9]. The BK and DK scores are divided into their quartile ranges. Further scores have been derived from the PKG that include the fluctuation score (FDS), percentage time immobile (PTI), and percent time with tremors (PTT). The PTI was defined as the percentage of 2-min periods between 9 AM and 6 PM, where the movement data recorded by the PKG device was very low and correlated with the daytime sleep measured by polysomnography (PSG) and the Epworth Sleepiness Scale Scores (ESS). The PTT was defined as the percentage of 2-min periods between 9 AM and 6 PM that contained tremor [24]. Tremor is likely to be present if PTT score is > 1%.

### Clinical measures

Clinical measures were obtained via telehealth within 2 weeks of use of the PKG [18]. The UPDRS was modified due to the limitations imposed by the telehealth methodology, in which assessments of rigidity and postural stability were excluded, similar to previous studies [19, 20]. The UPDRS was utilized due to its ability to assess both motor and non-motor aspects of PD symptoms. The UPDRS is divided into four categories: [1] mentation, behavior, and mood; [2] activities of daily living; [3] motor examination; and [4] complications of therapy. The sub-scales are rated from a scale of 0 (normal), 1 (slight), 2 (mild), 3 (moderate), and 4 (severe) [21].

The Abnormal Involuntary Movements Scale (AIMS) was developed to assess the occurrence of tardive dyskinesia in patients receiving neuroleptics but has been broadly validated to measure choreic movements in a range of neurodegenerative disorders [22]. It is a twelve-item scale divided into assessments of orofacial movements, extremity and truncal dyskinesias, and global severity and patient awareness. This was also assessed with a similar scoring system to the UPDRS of zero to four.

The Epworth Sleepiness Scale questionnaire, consisting of 8 questions (maximal score of 24), was used to assess daytime sleepiness [23]. We also utilized PDQ-8 scale, which includes 8 questions that are used to assess how often people affected by Parkinson’s experience difficulties across different dimensions of daily living, and is scored out of a maximum of 32 [24]. The Falls Efficacy Scale-International (FES-I), a measure of “fear of falling,” including 16 questions with a score range from 16 to 64 [25] was also utilized. Cognition was assessed with the NUCOG [26], a 5-domain scale that measures Attention, Visuospatial Function, Memory, Executive Function, and Language (Domains A–E) each out of 20, with a maximal score of 100.

### Data analysis

All analyses were performed in the R software (version 3.6.3; R Core Team, 2020 [27]). Outliers were identified with both Dixon tests and boxplots for visualization, and hence, various correlations depict less than 12 sample points. Pearson’s correlation coefficients were used to investigate the association between PKG parameters and other variables. Due to the sample size and skewed distribution of key variables, robust statistical methods were used. Specifically, bootstrapped bias corrected and accelerated (Bca) 95% confidence intervals were computed for all key analyses with 2000 replicates. Statistical significance was defined as 95% Bca confidence intervals that did not capture the null hypothesis value.

## Results

### Clinical characteristics

Clinical characteristics of the patients are shown in Supplementary Fig. 1 with the results depicted as mean (standard deviation). The sample consisted of 12 patients, with 6 (50%) females, and an average age of 38 (13.2). Antipsychotic use was present in 4 (33%), and 1 patient required a wheelchair with 3 others requiring one-person assistance for their mobility.

The median BKS score for the cohort of patients was 14.6 (6.6)–22.0 (7.4)–31.6 (10.2) (normative BKS scores: 12.7–18.6–26.1) and the median DKS score was 0.2 (0.8)–1.2 (3.1)–7.4 (7.8) (normative DKS scores: 0.9–4.3–16.5) (Table 1). Median FDS, PTS, and PTT scores were 8.1 (3.5), 4.9 (8.2), and 0.5 (0.6), respectively, whereas the normative scores are 10.8, 2%, and 1% respectively. UPDRS parts 1, 2, 3, and 4 and the UPDRS total respective scores were 2.5 (3.3), 8.5 (17.5), 16.5 (25.5), 1 [4], and 29.5 (41.8). The median PDQ score was 6 (6.8), AIMS 2.5 (14.8), Epworth Sleepiness Scale 6 (4.8), and Falls Efficacy Scale 20.5 (12.5). The median NUCOG parts A, B, C, D, and E and the total scores were 14.5 (5.8), 13 (4.1), 13 (10.9), 10.8 (8.3), 17.5 (7.3), and 68 (33.9).

**Table 1:** Overall characteristics of NPC cohort. Age is represented as mean (standard deviation). Data are then presented as number (%), or median (interquartile range). *PKG*, Personal KinetiGraph; *BKS*, bradykinesia score; *DKS*, dyskinesia score; *FDS*, fluctuation score; *PTI*, percentage time immobile; *PTT*, percentage time with tremor; *UPDRS*, Unified Parkinson’s Disease Rating Score; *PDQ*, Parkinson’s Disease Questionnaire; *AIMS*, Abnormal Involuntary Movement Scale; *ESS*, Epworth Sleepiness Score; *Falls*, Falls questionnaire; *NUCOG*, Neuropsychiatry Unit Assessment Tool

	Overall cohort	
No. of patients		12
Females		6 (50%)
Age (years)		38 (13.2)
Disease duration		9 (3.4)
Antipsychotic use		4 (33%)
Gait assistance		4 (33%)
PKG	Reference range	
BKS 25–50–75	12.7–18.6–26.1	14.6 (6.6)–22.0 (7.4)–31.6 (10.2)
DKS 25–50–75	0.9–4.3–16.5	0.2 (0.8)–1.2 (3.1)–7.4 (7.8)
FDS	10.8	8.1 (3.5)
PTI	2%	4.9% (8.2)
PTT	1%	0.5% (0.6)
UPDRS score		
UPDRS part I score		2.5 (3.3)
UPDRS part II score		8.5 (17.5)
UPDRS part III score		16.5 (25.5)
UPDRS part IV score		1 (4)
UPDRS total score		29.5 (41.8)
PDQ		6 (6.8)
AIMS		2.5 (14.8)
ESS		6 (4.8)
Falls		20.5 (12.5)
NUCOG score		
NUCOG – A score		14.5 (5.8)
NUCOG – B score		13 (4.1)
NUCOG – C score		13 (10.9)
NUCOG – D score		10.8 (8.3)
NUCOG – E score		17.5 (7.3)
NUCOG total score		68 (33.9)

### *BK scores and Falls correlation (Fig. **1**)*

**Fig. 1:**
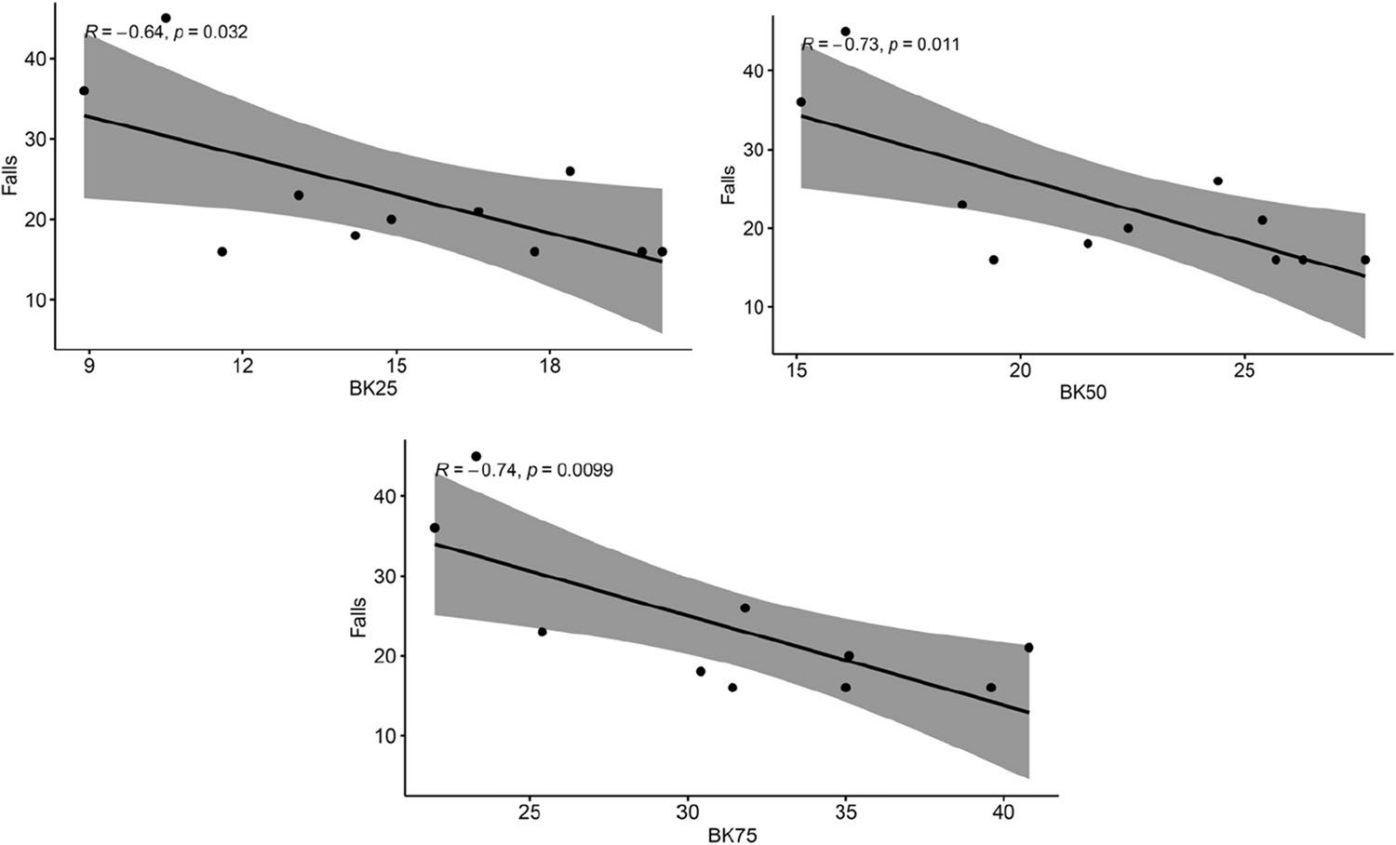
Bradykinesia scores (BK) plotted against Falls questionnaire. BK25, 25th percentile; BK50, 50th percentile; BK75, 75th percentile

Significant correlations were demonstrated between BK25 and Falls Efficacy Scale (*r* =  − 0.74, 95% CI = [− 0.95, − 0.08]), BK50 and Falls Efficacy Scale (*r* =  − 0.79, 95% CI = [− 0.96, − 0.19]), and BK75 and Falls Efficacy Scale (*r* =  − 0.76, 95% CI = [− 0.95, − 0.11]). Antipsychotic use did not confound the results.

### *FDS and multiple clinical correlations (Fig. **2**)*

**Fig. 2:**
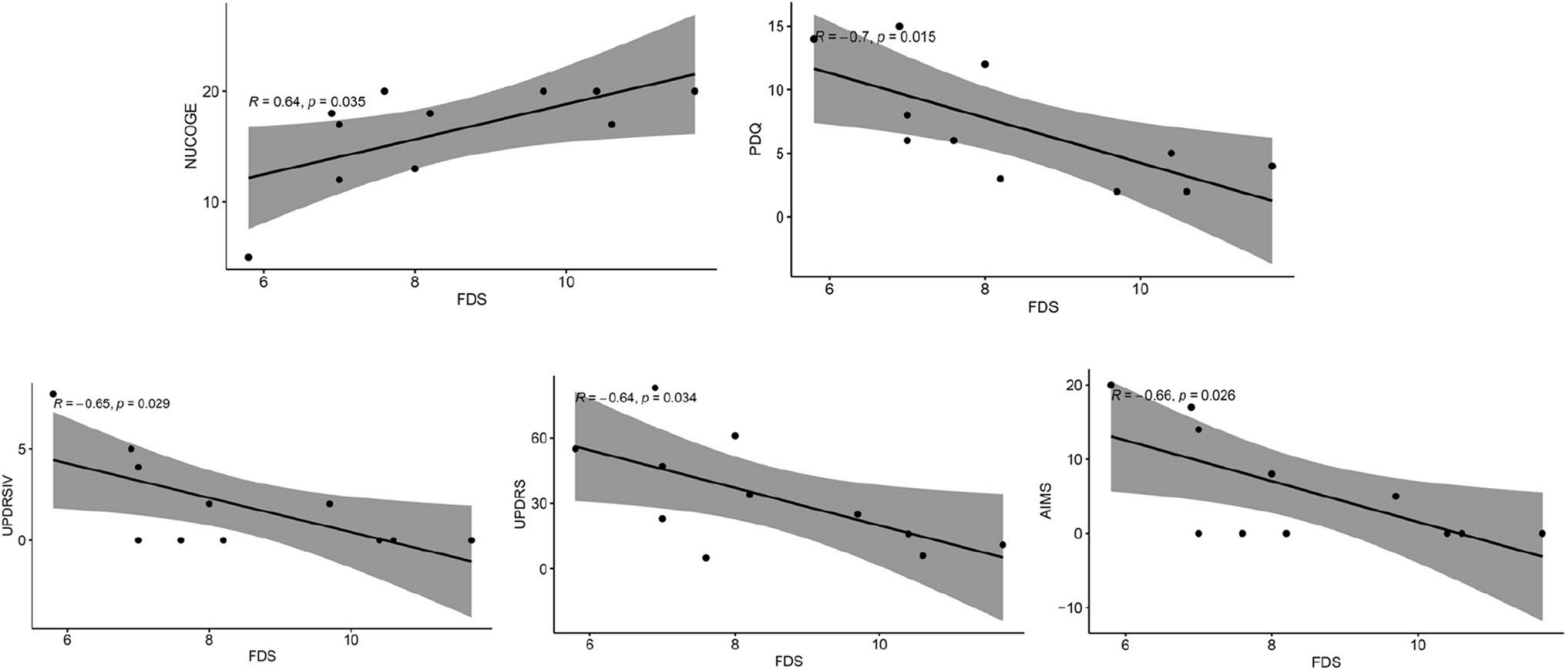
Fluctuation score (FDS) on comparison with Parkinson’s Disease Questionnaire (PDQ), Unified Parkinson’s Disease Rating Scale (UPDRS) part IV, UPDRS total score, and Abnormal Involuntary Movement Scale (AIMS)

The FDS score was compared to various clinical questionnaire parameters. Significant correlations were obtained in comparison to PDQ (*r* =  − 0.7, 95% CI = [− 0.92, − 0.18]), UPDRS IV (*r* =  − 0.65, 95% CI = [− 0.90, − 0.09]), UPDRS (*r* =  − 0.64, 95% CI = [− 0.9, − 0.06]), and AIMS (*r* =  − 0.96, 95% CI = [− 0.99, − 0.49]). Antipsychotic was a confounding variable, resulting in a strengthening of the relationship between FDS and UPDRS.

### *DK correlations with UPDRSI and NUCOGA (Fig. **3**)*

**Fig. 3:**
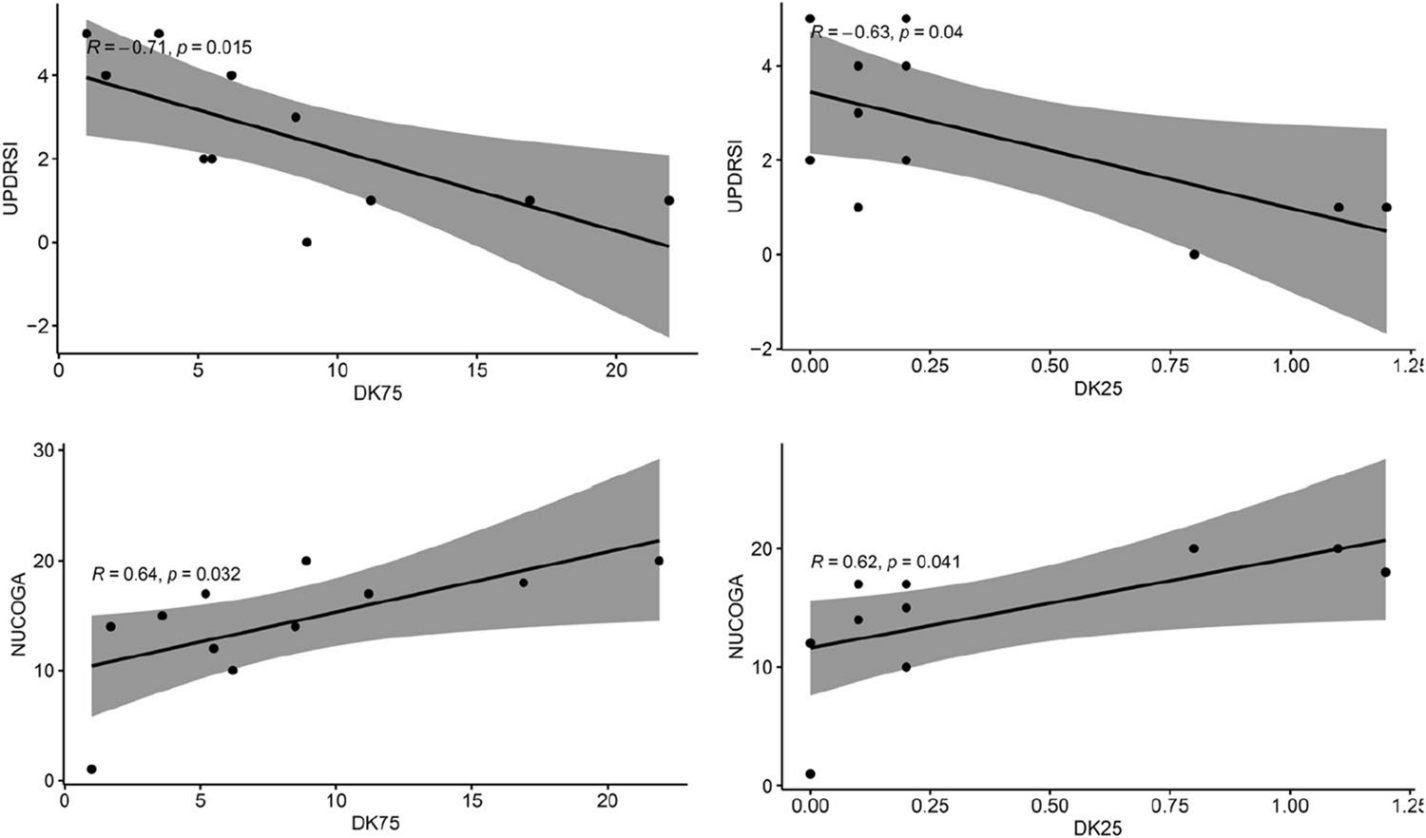
Dyskinesia score (DK) comparison with Unified Parkinson’s Disease Rating Scale (UPDRS) Part I and Neuropsychiatry Unit Assessment Tool – Attention (NUCOGA). DK25, 25th percentile; DK75, 75th percentile

DK25 in comparison with NUCOGA (*r* = 0.72, 95% CI = [0.17, 0.93]), and DK75 in comparison with NUCOGA (*r* = 0.63, 95% CI = [0.01, 0.90]) and NUCOG (*r* = 0.64, 95% CI = [0.02, 0.91]) demonstrated significant correlations. Antipsychotic use was a confounding variable that weakened the relationship between DK25/75 and NUCOG-A.

### *Duration and PTI (Fig. **4**)*

**Fig. 4:**
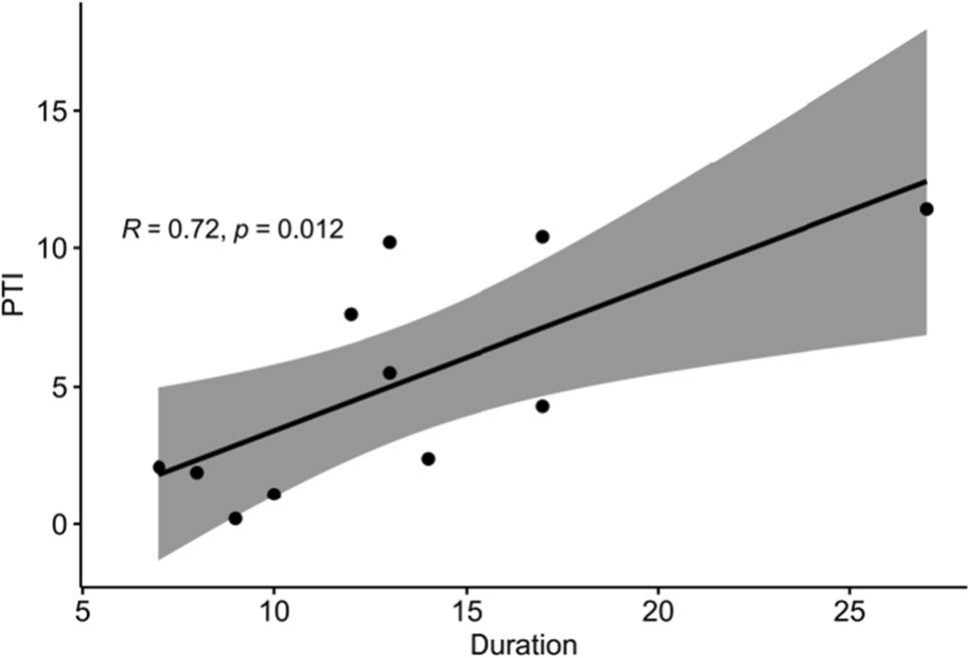
Duration of disease (years) in comparison to percentage time immobile (PTI)

Duration in comparison with PTI (*r* = 0.72, 95% CI = [0.22, 0.92]) demonstrated significance.

Confounders did not alter the result.

## Discussion

Neurological disorders characterize NPC and develop later during lifetime. They classically consist of ataxia, vertical supranuclear gaze palsy, dystonia, dysarthria, dysphagia, gelastic cataplexy, seizures, chorea, spasticity, and progressive dementia [5]. This was a small exploratory study aimed to assess the potential use of a continuous wearable movement sensor to better characterize movement disorders demonstrated within individuals with NPC. Furthermore, we attempted to correlate the output of the PKG with clinical assessments and questionnaires.

## DKS

There was a wide spectrum of clinical severity of movement disorders exhibited in our cohort of NPC patients. Surprisingly, hyperkinetic movements classically described in NPC patients were less severe as detected via the PKG in this cohort as compared to a standardized population (Table 1) [28]. Two patients (patients three and six) demonstrated elevated DKS scores, with patient six representing an outlier among the general cohort. However, a lack of a direct statistical comparison to a control group needs to be considered when making this observation.

The lack of correlation between the DKS and AIMS likely represents the differing neurobiology of levodopa-induced dyskinesia (LID) to which the PKG is most sensitive, and the chorea seen in NPC. Phenomenologically, LID in PD represents a hyperkinetic movement disorder that can be confused with, for example, moderately vigorous exercise on the PKG. In contrast, the chorea of NPC appears to be highly activation dependent and most evident during volitional movements and least at rest. Unsurprisingly, therefore, our cohort showed significant correlations between clinical scores of chorea (UPDRS part IV and AIMS) and the FDS instead, as the FDS refers to the *range* in fluctuations.

### DK correlations

DK 25th and 75th percentile scores correlated positively with the NUCOGA and negatively with the UPDRS part I (Fig. 3). The NUCOG-A assesses an individual’s attention, in which individuals with a high DKS performed better in this domain. It has previously been shown that attention and executive function are one of the first cognitive functions to be impaired in NPC [29, 30]. As the disease progresses, NPC patients’ cognition progressively deteriorates with manifestations in memory, language, visuospatial, and information processing domains. Hence, this correlation may indicate that the dyskinetic individuals may represent an earlier timepoint within the disease, and as the disease pathology progresses, they may generally move less (i.e., lower DKS), as attentional function worsens. However, antipsychotic use resulted in a weakened association between DK25/75 and NUCOG-A. Hence, individuals’ significant dyskinesias experiences may be partly explained by earlier antipsychotic use, and the effect that they represent an earlier timepoint in the disease. Additionally, our cohort performed worse in the executive domain of the NUCOG examination, which is consistent with previous studies [29, 30].

#### BKS

With a novel accelerometry device, we found bradykinesia to be a feature among this cohort of NPC patients. While it is not phenotypically classically recognized in NPC, it is likely clinically underestimated. Evidence within case reports have described bradykinesia on examination in patient diagnosed with NPC with prior incorrect labels of progressive supranuclear palsy and Parkinson’s [31]. A further case report of a 26-year-old female with typical movement disorders associated with NPC, as well as bradykinesia, was found to have a unilateral deficit on her striatal dopamine transporter scan [32]. Furthermore, a 52-year-old with bradykinetic finger tapping was found to have I-123-FP-CIT SPECT evidence of marked symmetrical loss of dopamine transport binding, especially within the putamen [33]. The co-existence of hyperkinesia’s and depressed voluntary motor activity in NPC may be explained by dysfunction of the indirect and direct striatal pathways.

### BK and falls

The BK score percentiles negatively correlated with the Falls Efficacy Scale-International (Fig. 1), demonstrating that as the BK scores increased, the NPC cohort scored lower on the Falls questionnaire. This suggests that the more bradykinetic an individual was, the less concern they reported with falling. This is in contrast to studies performed in Parkinson’s disease patients, in which clinical assessments of increased bradykinesia predicted an increased propensity for falls [34]. Bradykinetic individuals in this cohort were not prone to using gait aids; however, out of the individuals with regular antipsychotic use, 75% demonstrated elevated BKS scores. This may potentially explain the conflicting associations, whereby individuals more prone to psychosis and requiring antipsychotics may subsequently demonstrate less insight into their physical condition and propensity for reporting concerns regarding falls [35].

Clinical examinations and assessments were conducted over telehealth. A modified UPDRS has been shown to be reliable and valid among Parkinson’s patients through telehealth assessments [19]. The lack of correlation of the UPDRS clinical measures, with objective measures of bradykinesia using the PKG, could be explained by the potential for presence of hyperkinetic movements to confound the clinical assessment of bradykinesia. Moreover, the evidence demonstrates that bradykinesia may be more prevalent than appreciated.

### Sleep disorders

Sleep disorders have been rarely described within NPC [17, 36]. Limited evidence include chronic insomnia, probable and confirmed obstructive sleep apnea, REM sleep behavior disorders, restless leg syndrome, and excessive daytime sleepiness, which has been occasionally reported to as an initial symptom of the disease [36]. We attempted to characterize excessive daytime somnolence by the use of the Epworth Sleepiness Scale [37]. We found that our cohort of patients demonstrated a higher normal daytime sleepiness with a median score of 6. However, there was significant variation among the cohort with a quarter demonstrating mild/moderate excessive daytime sleepiness. Additionally, the PKG provides a percentage time immobile (PTI) score, which is a surrogate marker for sleep and has been validated in Parkinson’s patients [15]. We found that the duration of illness correlated positively with the PTI, hence indicating that the longer the disease process, the more immobile the individual. Additionally, the PTI score was elevated in our cohort, and hence, these findings are consistent with the findings within the literature regarding increased rates of daytime sleepiness in NPC patients.

### FDS correlations

The FDS, a measure of motor fluctuations, was produced by the summation of the interquartile ranges of the bradykinesia and dyskinesia scores and was noted to be able to distinguish between fluctuating and non-fluctuating patients with high sensitivity [12]. In the NPC cohort, the FDS was shown to negatively correlate with the UPDRS total score, UPDRS part IV, PDQ, and AIMS scores (Fig. 2). This demonstrates that individuals with low fluctuation may be predominantly hyperkinetic throughout the day with limited bradykinesia, as demonstrated by higher scores in the UPDRS part IV and AIMS scales. This trend supports the classic description of hyperkinetic movements among NPC patients as reported in the literature, however has yet to be quantified using a continuous wearable device [38]. Additionally, antipsychotic use was demonstrated as a significant confounder that strengthened this association, and hence, the continuous fluctuation may be partly due to this confounding variable.

### Limitations

This was an exploratory study examining a small cohort of individuals with limitations in the sample size, which is to be expected when studying a disease with a low prevalence. Furthermore, single point assessments of the clinical ratings and questionnaires represented further limitations in the interpretation of the results. This would have resulted in assessments capturing individuals when they are potentially fluctuating significantly throughout the day and hence not depicting their clinical status accurately. This could be improved by performing multiple clinical assessments at varying times of the days, which would also need to be completed by the same examiner/s to reduce examination variability. Due to the low prevalence of Niemann-Pick C, majority of the questionnaires are not validated in this cohort of patients and hence represent a further limitation of this study. Additionally, the PKG does not measure the amplitude of the tremor nor acceleration of movement, which may make differentiation of involuntary from voluntary movements difficult. Lastly, the PKG has been most studied and optimized to detect movement disorders associated with Parkinson’s disease and its management. It is possible with a larger cohort that the movement algorithms could be modified to better measure the abnormal movements of NPC.

### Future directions

The low prevalence of Niemann-Pick C represents difficulty studying reported movement disorders within this cohort of patients. This study allowed objective assessments of various parameters that assist in the understanding of the natural day-to-day fluctuations of the movement disorders experienced by these patients. Further studies will need to utilize larger sample sizes and could also examine the natural history of these movement disorders and correlate them with disease severity and progression.

## Conclusion

The use of the PKG demonstrated that bradykinesia is under recognized among NPC patients and that bradykinetic patients were less likely to report concerns regarding falls. Additionally, the FDS rather than the DKS is sensitive to the abnormal involuntary movements of NPC—reflecting a differing neurobiology of this type of chorea compared to levodopa-induced dyskinesia. In this cohort, DKS was inversely correlated with measurements of cognition suggesting that with cognitive decline, movement in general declines.
